# Oxygen Pressure Influence on Properties of Nanocrystalline LiNbO_3_ Films Grown by Laser Ablation

**DOI:** 10.3390/nano10071371

**Published:** 2020-07-14

**Authors:** Zakhar Vakulov, Evgeny Zamburg, Daniil Khakhulin, Andrey Geldash, Dmitriy A. Golosov, Sergey M. Zavadski, Andrey V. Miakonkikh, Konstantin V. Rudenko, Anatoliy P. Dostanko, Zhubing He, Oleg A. Ageev

**Affiliations:** 1 Federal Research Centre The Southern Scientific Centre of the Russian Academy of Sciences (SSC RAS), 41 Chekhov St., 344006 Rostov-on-Don, Russia; 2 Department of Electrical & Computer Engineering, National University of Singapore, 4 Engineering Drive 3, Singapore 117583, Singapore; zamburg@nus.edu.sg; 3 Research and Education Centre ‘Nanotechnologies’, Southern Federal University, 2 Shevchenko St., 347922 Taganrog, Russia; dhahulin@sfedu.ru (D.K.); ageldash@sfedu.ru (A.G.); ageev@sfedu.ru (O.A.A.); 4 Department of Electronic Technology and Engineering, Belarusian State University of Informatics and Radioelectronics, 6 P. Brovki Str., 220013 Minsk, Belarus; dmgolosov@mail.ru (D.A.G.); szavad@bsuir.by (S.M.Z.); kafett@bsuir.by (A.P.D.); 5 Laboratory of Microstructuring and Submicron Devices, Valiev Institute of Physics and Technology of the Russian Academy of Sciences, 36/1 Nakhimovsky Av., 117218 Moscow, Russia; miakonkikh@ftian.ru (A.V.M.); rudenko@ftian.ru (K.V.R.); 6 Department of Materials Science and Engineering, Shenzhen Key Laboratory of Full Spectral Solar Electricity Generation (FSSEG), Southern University of Science and Technology, 1088 Xueyuan Rd., Shenzhen 518055, Guangdong, China; hezb@sustc.edu.cn

**Keywords:** pulsed laser deposition, lithium niobate, thin films, lead-free energy conversion devices

## Abstract

Energy conversion devices draw much attention due to their effective usage of energy and resulting decrease in CO_2_ emissions, which slows down the global warming processes. Fabrication of energy conversion devices based on ferroelectric and piezoelectric lead-free films is complicated due to the difficulties associated with insufficient elaboration of growth methods. Most ferroelectric and piezoelectric materials (LiNbO_3_, BaTiO_3_, etc.) are multi-component oxides, which significantly complicates their integration with micro- and nanoelectronic technology. This paper reports the effect of the oxygen pressure on the properties of nanocrystalline lithium niobate (LiNbO_3_) films grown by pulsed laser deposition on SiO_2_/Si structures. We theoretically investigated the mechanisms of LiNbO_3_ dissociation at various oxygen pressures. The results of x-ray photoelectron spectroscopy study have shown that conditions for the formation of LiNbO_3_ films are created only at an oxygen pressure of 1 × 10^−2^ Torr. At low residual pressure (1 × 10^−5^ Torr), a lack of oxygen in the formed films leads to the formation of niobium oxide (Nb_2_O_5_) clusters. The presented theoretical and experimental results provide an enhanced understanding of the nanocrystalline LiNbO_3_ films growth with target parameters using pulsed laser deposition for the implementation of piezoelectric and photoelectric energy converters.

## 1. Introduction

Over the past few decades, the range of wireless wearable sensors and portable electronic devices has expanded significantly, and in most cases, their power supply is provided by electrochemical batteries [1,2,3]. Although the performance of electronic devices is increasing year on year, significant progress in improving the efficiency of batteries has not been achieved yet [4]. In search of a solution to this issue, companies and research teams around the world are considering the possibility of obtaining electric power from the environment. There are many sources of energy: mechanical, thermal, chemical, and solar, that can be converted into electrical energy [5]. Piezoelectric materials are widely used in the design and manufacture of energy converters that enable an effective conversion of mechanical energy of deformations (vibrations) into an electric current [6,7,8].

The possibility of creating miniature piezoelectric energy converters opens up wide opportunities for their integration with “smart clothes” and wearable electronic devices, thus leading to the demand for using lead-free materials [9]. In addition, particular successes in creating highly efficient piezoelectric energy converters are associated with using carbon nanotubes (CNTs) and the modification of their properties by deposition of piezoelectric materials on their surface or using CNTs as part of a piezoelectric nanocomposite [10,11]. Hence, composites and ferroelectric films are promising materials for the fabrication of piezoelectric energy converters.

Due to the unique combination of physical and chemical properties, ferroelectric materials are widely used in the production of integrated optical devices, waveguide structures, phase modulators, piezoelectric transducers, and surface acoustic waves devices [12,13,14,15]. Besides, ferroelectric materials are promising for photoelectric converters due to their charging properties [16]. However, in most cases, piezoelectric films are based on multicomponent oxides (BaTiO_3_, SrTiO_3_, LiNbO_3_), and their properties are determined by the stoichiometric composition and structure, which depend on the growth method and fabrication parameters. Combined with the insufficiently developed multicomponent oxide films, fabrication technology results in the need to study the influence of growth conditions on properties of ferroelectric films.

One of the promising materials for piezoelectric energy converters is LiNbO_3_, since it has high piezoelectric efficiency and Curie temperature, and meets environmental safety requirements (i.e., does not contain lead) [17]. Various technological methods are being used for LiNbO_3_ films formation: epitaxy [18], chemical vapor deposition [19], reactive magnetron sputtering [20], sol-gel process [21], and pulsed laser deposition (PLD) [22,23,24,25]. The latter has shown a substantial prospective for the fabrication of multi-component inorganic films [26] since it enables the control of a number of technological parameters and makes it possible to obtain films with controlled properties [27]. The background pressure largely determines the composition and properties of films growing by PLD [28]. Moreover, in the last 15 years, the study of LiNbO_3_ films has focused on their possible integration with planar technology to create new devices [29]. Despite a large number of publications on the synthesis of LiNbO_3_ films and the possibility of creating various functional devices based on them, the practical use of LiNbO_3_ films in micro- and nanoelectronics is significantly limited due to the lack of a compatible technology for the fabrication of thin ferroelectric films. Using buffer layers allows us to obtain films with a higher degree of crystallinity [30]. Nevertheless, the use of additional layers for LiNbO_3_ film synthesis can significantly complicate the fabrication process and can limit the possibilities for their subsequent heat treatment. The SiO_2_/Si structure is one of the most attractive substrates for the deposition of LiNbO_3_ films since it enables the direct integration of piezoelectric converters with sensitive elements of wireless wearable sensors and portable electronics [31].

Recently, a large number of publications have been devoted to the synthesis of lithium niobate films and structures based on it [32,33,34,35]. However, requirements for the structure, morphology, and properties of LiNbO_3_ films are formulated only in the ‘first approximation’ [29]. Summarizing, an urgent problem is the production of nanocrystalline piezoelectric films with controlled parameters. This work presents the results of a complex study that provides the regularities of LiNbO_3_ films formation by PLD. The purpose of the experimental studies is to determine the influence of oxygen pressure on the properties of LiNbO_3_ films grown by PLD. In order to study the phase formation processes, the analysis of possible chemical reactions in the system of lithium-niobium-oxygen materials is carried out by calculating and analyzing the temperature dependencies of the change in Gibbs free energy considering deposition modes.

## 2. Materials and Methods

### 2.1. Thermodynamic Simulation

Laser ablation includes complex non-stationary processes: fast heating, overheating, and rapid nucleation. The description of the thermal mechanisms of laser ablation (surface evaporation, homogeneous boiling, and phase explosion) is a complex task and is accurately described in terms of non-equilibrium thermodynamics [36]. However, for preliminary theoretical estimates, we used equilibrium thermodynamics approaches based on the calculation of the Gibbs free energy temperature dependence.

It can be assumed that the ablated LiNbO_3_ target can dissociate into individual components since the temperature at the interaction region of laser radiation with the target surface usually reaches several thousand degrees Celsius and significantly exceeds the LiNbO_3_ melting temperature [37,38].

In order to study the processes related to the dissociation of LiNbO_3_, it is essential to determine possible dissociation reactions of lithium niobate by calculating and analyzing temperature dependences of change in Gibbs free energy (Δ*G*) considering the nonlinear temperature dependences of the thermo-physical properties of materials [39]:

(1) ΔG(T)=ΔH−TΔS,

where Δ*H* and Δ*S* – change of enthalpy [J/mol] and entropy [J/K] of a reaction, *T* – temperature [K].

The temperature dependences of the change in Gibbs free energy are calculated using the FactSage 6.2 software package for chemical reaction analysis (GTT-Technologies, Herzogenrath, Germany), which has a regularly updated electronic database of temperature dependences of the materials’ thermophysical parameters. Calculating Δ*G*, we take into account not only the possibility of interaction between the components (for example, lithium and niobium oxides can interact with oxygen formed as a result of LiNbO_3_ dissociation) but also the influence of background pressure in the growth chamber. Such calculations allow us to promptly evaluate the optimal window of partial oxygen pressures, as well as temperature.

In order to analyze the effect of oxygen pressure on the LiNbO_3_ dissociation reactions, the following decomposition reactions of lithium niobate in a vacuum (1 × 10^−5^ Torr) and oxygen atmosphere (1 × 10^−2^ Torr) are identified:

(2) $$LiNbO_{3}\to Li_{2}O+NbO+O_{2},$$

(3) $$LiNbO_{3}\to Li_{2}O+NbO_{2}+O_{2},$$

(4) $$LiNbO_{3}\to Li_{2}O+Nb_{2}O_{5},$$

(5) $$LiNbO_{3}\to Li_{2}O_{2}+NbO+O_{2},$$

(6) $$LiNbO_{3}\to Li_{2}O_{2}+NbO_{2},$$

(7) $$LiNbO_{3}\to Li+Nb+O_{2}.$$

Stoichiometric coefficients in the equations of chemical reactions are taken into account, but omitted here, in order to simplify the perception of the results.

### 2.2. Experimental Methods

To synthesize LiNbO_3_ films, we use the nanotechnological cluster complex NANOFAB NTK-9 (NT-MDT, Zelenograd, Russia), comprising the PLD module Pioneer 180 (Neocera Co., Beltsville, MD, USA). LiNbO_3_ congruent target (Kurt J. Lasker, 99.9% purity) is ablated by excimer KrF laser (λ = 248 nm) (Coherent Inc., Santa Clara, CA, USA). Energy density on the target surface is maintained at 1.5 J/cm^2^. In all experiments, the target-substrate distance (100 mm), number of pulses (50,000), pulse repetition rate (10 Hz), and laser pulses energy on the target surface (150 mJ) are kept constant. Background oxygen pressure in the growth chamber varied from 1 × 10^−5^ Torr to 1 × 10^−2^ Torr. Films are obtained with a thickness of 45–90 nm at the heater temperature of 600 °C on SiO_2_ (100 nm)/Si structures. The effect of the SiO_2_ buffer layer thickness on the morphological parameters of LiNbO_3_ films is presented in [40].

The morphology of the obtained films is studied by scanning electron microscopy (SEM) and atomic force microscopy (AFM) in semi-contact mode using a Nova Nanolab 600 scanning electron microscope (FEI. Co., Eindhoven, the Netherlands) and a Ntegra probe nanolaboratory (NT-MDT, Zelenograd, Russia), respectively. The crystal structure and elemental composition of the obtained LiNbO_3_ films are studied by X-ray diffraction (XRD) and X-ray photoelectron spectroscopy (XPS) using Rigaku MiniFlex 600 (Rigaku Co., Tokyo, Japan) and Kratos Axis Ultra X-ray Photoelectron Spectroscopy (XPS) instrument (Kratos Analytical Ltd., Manchester, UK), respectively. XPS spectra were analyzed using the OPUS 7.0 software (Bruker Co., Billerica, MA, USA). The charge carriers concentration and mobility are determined by measuring the Hall electric moving force using an Ecopia HMS-3000 measurement system (Ecopia Co., Anyang, Republic of Korea). The spectral dependencies of the optical characteristics (refractive index *n* and absorption coefficient *k*) are studied on spectral ellipsometer M-2000X (J.A. Woollam Co., Lincoln, NE, USA) under the beam angle of 65° in the wavelength range from 240 nm to 1000 nm with 10-nm pitch. The spot size is about 2 × 5 mm.

## 3. Results and Discussion

### 3.1. Theoretical Results

**Figure 1:**
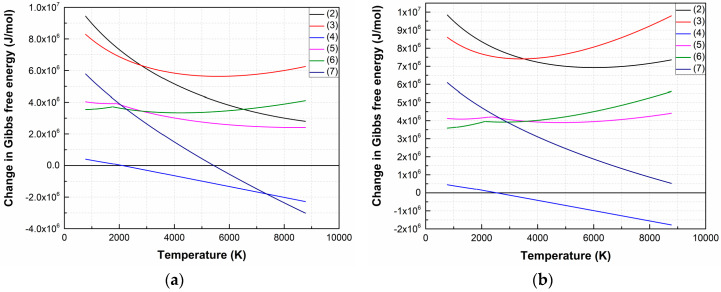
Δ*G* temperature dependences of LiNbO_3_ decomposition reactions in a vacuum (1 × 10^−5^ Torr) (**a**) and oxygen atmosphere (1 × 10^−2^ Torr) (**b**).

Figure 1 shows the temperature dependences Δ*G* of LiNbO_3_ dissociation reactions in vacuum and oxygen atmosphere. The temperature range is determined by the temperatures of the laser plume and the substrate (maximum and minimum temperatures, respectively), based on the data presented in the literature [37] and the theoretical estimation of the laser plume parameters according to [41].

Analysis of the dependences has shown that the most probable dissociation reaction is (4) both for vacuum (1 × 10^−5^ Torr) and oxygen atmosphere (1 × 10^−2^ Torr), which occurs at temperatures above 2113 K and 2533 K, respectively. Dissociation of LiNbO_3_ into individual elements (7) is possible when the temperature increased to 5443 K (in a vacuum).

**Figure 2:**
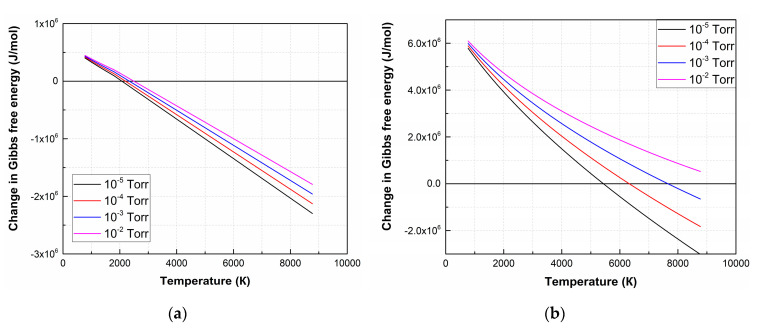
Δ*G* temperature dependences of the decomposition reactions of LiNbO_3_ into Li_2_O and Nb_2_O_5_ (4) (**a**) and individual elements (7) (**b**) at different oxygen pressures.

The Δ*G* value of the remaining reactions is positive in the entire temperature range at an oxygen pressure of 1 × 10^−2^ Torr, hence the forward direction of the reaction is impossible in the temperature range from 773 K to 8773 K. Figure 2 shows temperature dependences Δ*G* of (4) and (7) at various oxygen pressures.

As a result of the analysis of thermodynamic regularities, it is found that the LiNbO_3_ dissociation is a multi-stage process, depending on temperature and the value of oxygen pressure. At the first stage, lithium niobate dissociates into oxides with lower oxides (Li_2_O, Nb_2_O_5_). According to Δ*G* analysis of Li_2_O, Nb_2_O_5_ decomposition reactions show that the oxides decompose and completely dissociate into Li, Nb, and O_2_ at temperatures above 2050 K [42]. At pressure 1 × 10^−2^ Torr, (7) becomes impossible, and LiNbO_3_ decompose into Li_2_O and Nb_2_O_5_ according to (4) [43]. With the subsequent propagation of laser plume toward the substrate, its temperature decreases, and the conditions for reverse reactions of Li and Nb interaction with O_2_ are created, as well as the formation of their oxides and lithium niobate.

### 3.2. Experimental Results

**Figure 3:**
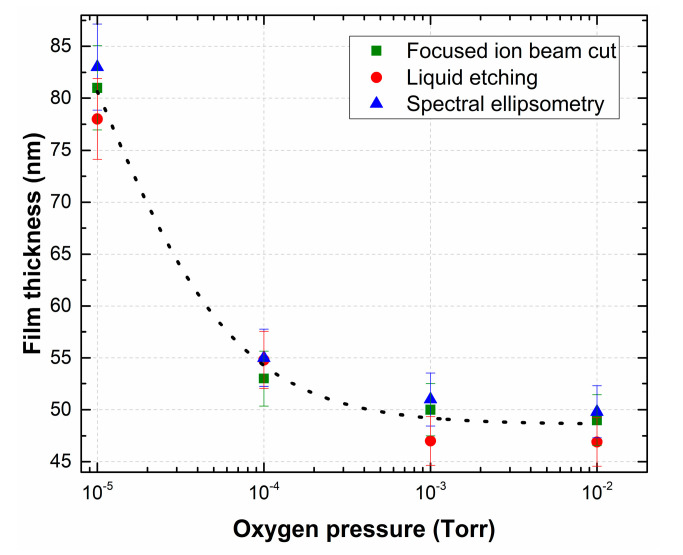
Dependence of the thickness of the LiNbO_3_ film as a function of oxygen pressure measured by different methods.

Figure 3 shows the dependence of the LiNbO_3_ film thickness on oxygen pressure measured by different methods.

We applied three mutually independent methods to measure the thickness of LiNbO_3_ films: (1) the focused ion beam cut; (2) liquid etching [44]; (3) spectral ellipsometry [45]. In addition, with increasing oxygen pressure in the growth chamber, the character of the plasma interaction in the laser plume changes, which causes phase formation and mass transfer during PLD [46]. A decrease in the film growth rate might be associated with decreasing of the ablated particles mean free path in the transit space under increasing background pressure in the growth chamber. The experimental results show that the thickness of LiNbO_3_ films decreases from 80.7 ± 7.8 nm (film deposition rate 0.9 nm/min) to 48.57 ± 3.5 nm (film deposition rate 0.54 nm/min) with the increase of oxygen pressure from 1 × 10^−5^ Torr to 1 × 10^−2^ Torr (Figure 3).

The films obtained at residual and oxygen pressures of 1 × 10^−5^ Torr and 1 × 10^−2^ Torr, respectively, are chosen for XPS studies since they are characterized by a change in the mechanism of LiNbO_3_ dissociation (Figure 2).

**Figure 4:**
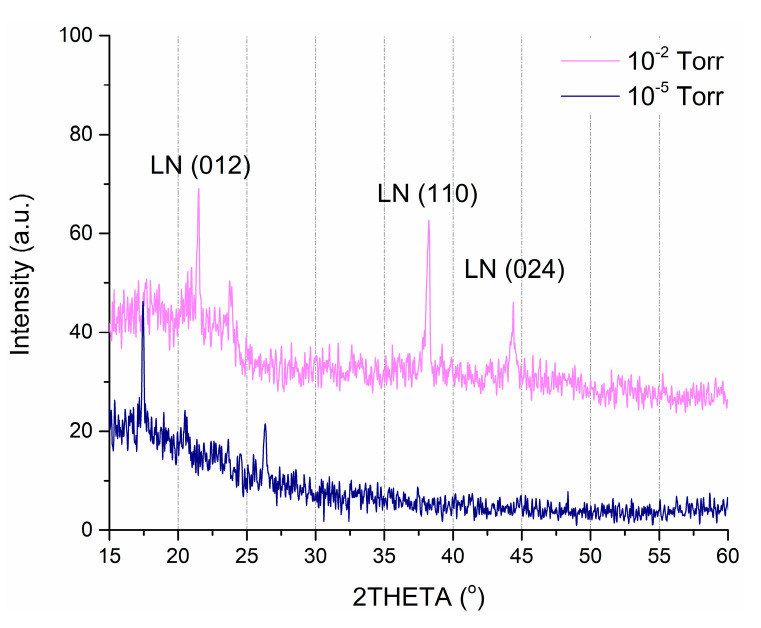
X-ray diffraction (XRD) spectra of LiNbO_3_ films, fabricated at an oxygen pressure of 1 × 10^−2^ Torr and 1 × 10^−5^ Torr.

The results of XRD analysis show that all obtained films have the nanocrystalline structure with the predominance of crystallites oriented in the (012), (110), and (024) planes (Figure 4). Figure 4 shows a comparison of the XRD spectra of the films deposited at an oxygen pressure of 1 × 10^−2^ Torr and 1 × 10^−5^ Torr.

We excluded angles higher than 60° since peaks in that region are attributed to the substrate. In the case of the higher pressure (1 × 10^−2^ Torr), two distinct reflections corresponding to the (012) and (110) crystal planes in the range from 15° to 60°. Moreover, this film has a single-phase structure, identified as the ferroelectric structure of bulk material from the group (R3c) LiNbO_3_ [47]. In comparison, lower pressure (1 × 10^−5^ Torr) sample shows peaks that correspond to lithium and niobium oxides while showing no presence of LiNbO_3_.

**Figure 5:**
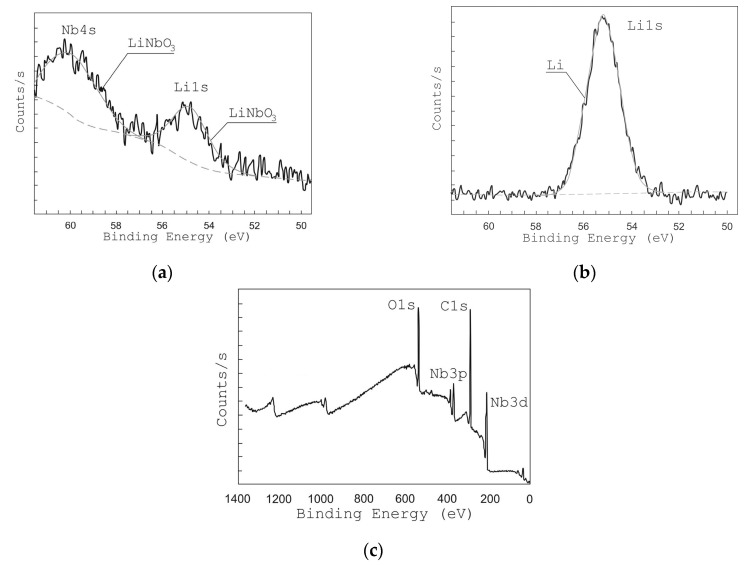
X-ray photoelectron spectroscopy (XPS) peaks from Li 1s and Nb 4s of films grown at different oxygen pressures: 1 × 10^−2^ Torr (**a**) and 1 × 10^−5^ Torr (**b**), and survey XPS spectra of LiNbO_3_ film (**c**).

In order to study the chemical bonds of the grown films, XPS analysis has been used. Figure 5c shows the XPS survey spectra of LiNbO_3_ films. The obtained spectra show lines corresponding to Li 1s, Nb 4s, Nb 3d, Nb 3p, and O 1s bonds [48].

**Figure 6:**
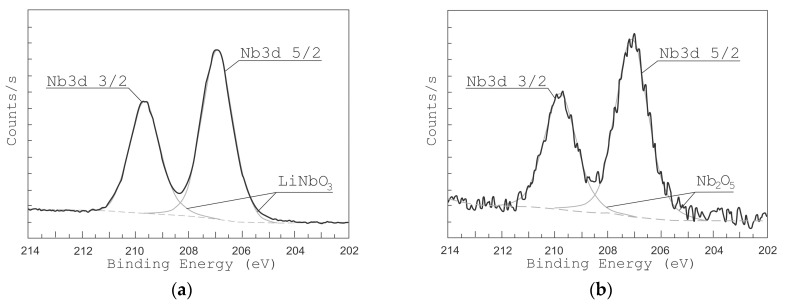
XPS peaks from Nb of LiNbO_3_ films grown at different oxygen pressures: 1 × 10^−2^ Torr (**a**) and 1 × 10^−5^ Torr (**b**).

Figure 5 and Figure 6 show the high-resolution XPS spectra of Li 1s and Nb 4s as well as Nb 3d lines for the grown LiNbO_3_ films. In the range of binding energies from 50 to 62 eV and from 202 to 214 eV, respectively, Li 1s, Nb 4s, and Nb 3d 3/2, Nb 3d 5/2 peaks are identified. To define various states of lithium and niobium atoms the peaks were decomposed by Gaussian functions [49,50,51]. XPS peaks from Nb 3d are decomposed into 3d 3/2 and 3d 5/2 contributions: NbO (207.47 and 204.67 eV) [51], NbO_2_ (208.48 and 205.68 eV) [51], Nb_2_O_5_ (209.84 and 207.16 eV) [51], and LiNbO_3_ (209.41 eV and 206.63 eV) [49,50]. Considering the contributions of LiNbO_3_ (54.8 eV, 60.2 eV) and Li_2_CO_3_ (55.2 eV) bonds, the XPS peaks of Li 1 s and Nb 4 s were decomposed. Peaks of other Li and Nb compounds in the range from 50 eV to 62 eV are absent.

The analysis of XPS spectra in the range from 50 eV to 62 eV shows that under residual pressure of 1 × 10^−5^ Torr (Figure 5b), the spectrum had only one Li 1s peak (55.2 eV), which attributes to Li_2_CO_3_. The Nb 4s peak corresponding to the LiNbO_3_ phase was absent. The peaks of Nb 3d 3/2 and Nb 3d 5/2 (Figure 6b) had maximum energy of 207.055 eV and 209.869 eV, respectively, which corresponds to Nb_2_O_5_ chemical bonds. The peaks corresponding to NbO, NbO_2_, and LiNbO_3_ are not detected.

The position of the peaks changed when oxygen is added during the film deposition (pressure 1 × 10^−2^ Torr). At this pressure, films show XPS peaks at 206.89 eV and 209.635 eV corresponding to LiNbO_3_ bonds while no peaks attributed to other types of bonds are detected. In the range of binding energies from 50 to 62 eV, one can see two peaks Li 1s (54.8 eV) and Nb 4s (60.2 eV), which corresponds to LiNbO_3_ bonds (Figure 5a) [49,50,51]. Similarly, the peaks of Nb 3d 3/2 and Nb 3d 5/2 (Figure 6a) had a maximum of 206.89 eV and 209.635 eV, which corresponds to the binding energy to LiNbO_3_ bonds.

**Figure 7:**
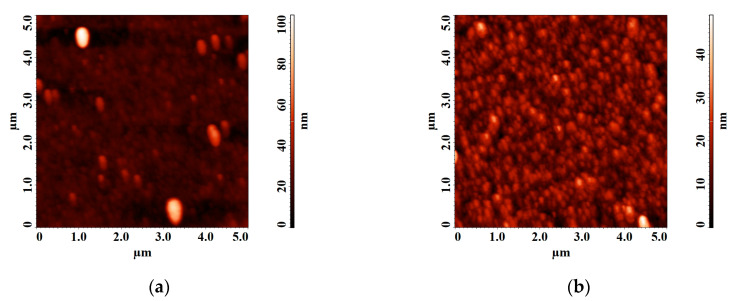
Atomic force microscopy (AFM) images of LiNbO_3_ film grown by pulsed laser deposition (PLD) at different oxygen pressures: 1 × 10^−5^ Torr (**a**), 1 × 10^−2^ Torr (**b**).

**Figure 8:**
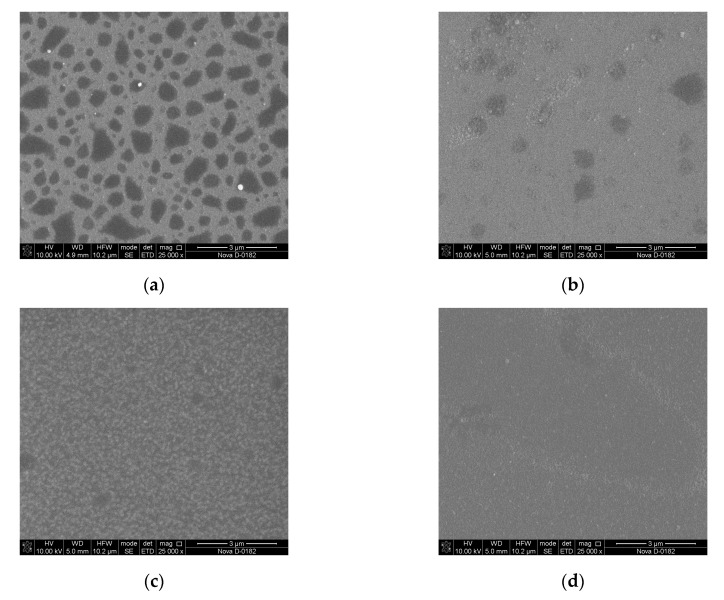
Scanning electron microscopy (SEM) images of LiNbO_3_ films grown by PLD at different oxygen pressures: 1 × 10^−5^ Torr (**a**), 1 × 10^−4^ Torr (**b**), 1 × 10^−3^ Torr (**c**), 1 × 10^−2^ Torr (**d**).

Based on the results of XPS analysis, we can conclude that the films grown at 1 × 10^−5^ Torr form a mixture of niobium oxide Nb_2_O_5_ and Li_2_CO_3_, which is associated with a lack of oxygen in the deposited film. This fact is confirmed by the results of SEM and AFM studies (Figure 7 and Figure 8). The decomposition of the multi-component material into individual atoms takes place during the ablation of LiNbO_3_ in the laser plume (7), and part of the oxygen atoms are pumped out by the pumping system. The lack of oxygen in the deposited film is compensated for, when oxygen is added to the chamber (pressure 1 × 10^−2^ Torr), which leads to the formation of a single-phase structure of LiNbO_3_.

It is established that the diameter of clusters on the surface of the film obtained at a residual pressure of 1 × 10^−5^ Torr is 92 ± 7.4 nm. With increasing oxygen pressure from 1 × 10^−5^ Torr to 1 × 10^−2^ Torr, the average roughness of the obtained films decreasing from 4.75 nm to 4.58 nm. Teardrop-shaped structures on the surface of the obtained films were identified as Nb_2_O_5_ [52,53].

**Figure 9:**
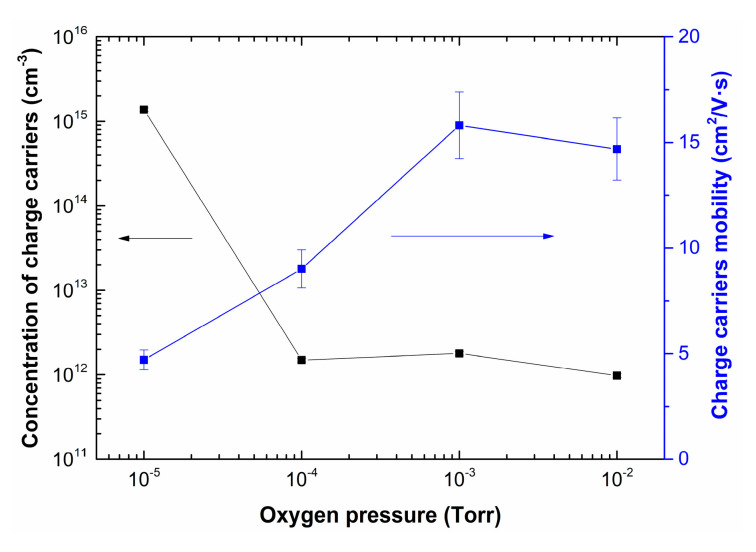
Dependences of concentration and charge carrier mobility of LiNbO_3_ films grown by PLD as a function of oxygen pressure.

Figure 9 shows the dependences of concentration and charge carrier mobility of LiNbO_3_ films as a function of oxygen pressure.

Increasing oxygen pressure from 1 × 10^−5^ Torr to 1 × 10^−2^ Torr results in decreasing of charge carrier concentration in the range from 1.4 × 10^15^ cm^−3^ to 9.7 × 10^11^ cm^−3^. In contrast, the mobility of charge carriers increased from 4.7 cm^2^/(V∙s) to 16 cm^2^/(V∙s). It is assumed that the electron mobility can change with changing the stoichiometry of LiNbO_3_: congruent LiNbO_3_ (Li to Nb ratio of about 94%) has lower electron mobility than a perfectly stoichiometric crystal (Li to Nb ratio is 1) [54]. This effect can be associated with changes in the phase composition of LiNbO_3_ films, as well as a decrease in the content of metallic Li and the defectiveness of the films, which is confirmed by the results of XPS, SEM, and AFM studies (Figure 5, Figure 6, Figure 7 and Figure 8).

**Figure 10:**
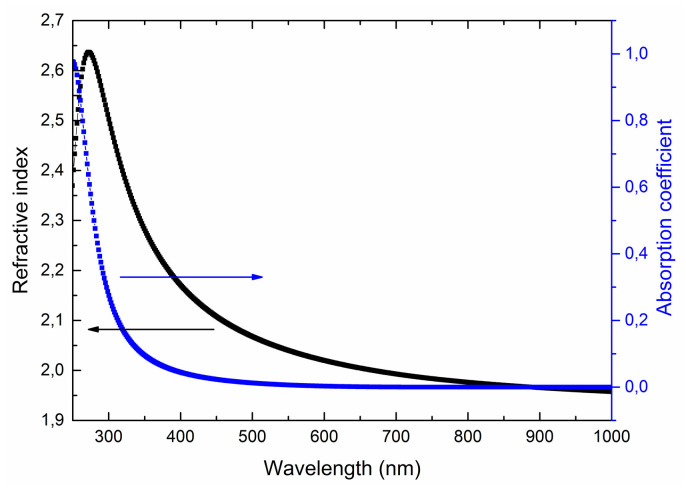
Spectral dependences of refractive index *n* and absorption coefficient *k* on a wavelength for the LiNbO_3_ films grown at oxygen pressure 1 × 10^−2^ Torr.

Figure 10 shows typical spectral dependences of the optical constants of LiNbO_3_ films on wavelength. The Tautz-Lorentz model [55] (which applies both to dielectrics and semiconductors) is used for modeling the optical characteristics of the films.

Obtained optical characteristics satisfy the Kramers-Kronig relations [56], and the film thicknesses are similar to the data obtained by the focused ion beam cut and liquid etching. It was found that the measurement results do not depend on the orientation of the samples, which indicates the isotropic nature of the optical characteristics of the obtained films. In the visible wavelength range, the refractive index decreases from 2.63 (at 350 nm) to 1.95 (at 800 nm). The absorption coefficient does not exceed 0.01. There is a slight decrease in the refractive index and a sharp increase in the absorption coefficient to 0.97 in the near-ultraviolet region of the spectrum.

## 4. Conclusions

Studies of the properties of LiNbO_3_ films grown by the PLD show that increasing oxygen pressure in the growth chamber has a significant effect on target dissociation mechanism, structure, composition, and properties of the deposited films. The results obtained by the theoretical assessment of thermodynamic processes show good agreement with the experimental data in the considered window of partial oxygen pressures and temperatures.

Analysis of XPS spectra shows that the formation of LiNbO_3_ films is possible at an oxygen pressure of 1 × 10^−2^ Torr. The films grown at residual pressure 1 × 10^−5^ Torr do not contain sufficient oxygen to form LiNbO_3_, which leads to the formation of Nb_2_O_5_ clusters on the films’ surfaces.

It was discovered that the structure of the films becomes more fine-grained, and the mobility of charge carriers increases from 4 cm^2^/V∙s to 16 cm^2^/V∙s with the increase of oxygen pressure from 1 × 10^−5^ Torr to 1 × 10^−2^ Torr. The refractive index of the obtained films ranges from 1.95 to 2.05 depending on the wavelength (60–800 nm), and the absorption index does not exceed 0.01.

The study shows the possibility of fabrication of LiNbO_3_ films with target properties by PLD. The obtained theoretical and experimental results make it possible to get LiNbO_3_ films that can be used for the fabrication of promising lead-free energy converters for “green” energy devices.
